# High Prevalence of Multidrug-Resistant *Clostridioides difficile* Following Extensive Use of Antimicrobials in Hospitalized Patients in Kenya

**DOI:** 10.3389/fcimb.2020.604986

**Published:** 2021-02-08

**Authors:** Winnie C. Mutai, Marianne W. Mureithi, Omu Anzala, Gunturu Revathi, Brian Kullin, Magdaline Burugu, Cecilia Kyany’a, Erick Odoyo, Peter Otieno, Lillian Musila

**Affiliations:** ^1^ Department of Medical Microbiology, School of Medicine, University of Nairobi, Nairobi, Kenya; ^2^ Department of Pathology, Division of Medical Microbiology, Aga Khan University Hospital, Nairobi, Kenya; ^3^ Department of Molecular and Cell Biology, Faculty of Science, University of Cape Town, Cape Town, South Africa; ^4^ US Army Medical Research Directorate-Africa, Nairobi, Kenya

**Keywords:** *Clostridioides difficile*, antimicrobial use, multidrug resistance, toxin types, Africa

## Abstract

**Introduction:**

*Clostridioides difficile* is a neglected pathogen in many African countries as it is generally not regarded as one of the major contributors toward the diarrheal disease burden in the continent. However, several studies have suggested that *C. difficile* infection (CDI) may be underreported in many African settings. The aim of this study was to determine the prevalence of CDI in hospitalized patients, evaluate antimicrobial exposure, and detect toxin and antimicrobial resistance profiles of the isolated *C. difficile* strains.

**Methods:**

In this cross-sectional study, 333 hospitalized patients with hospital-onset diarrhoea were selected. The stool samples were collected and cultured on cycloserine-cefoxitin egg yolk agar (CCEY). Isolates were presumptively identified by phenotypic characteristics and Gram stain and confirmed by singleplex real-time PCR (qPCR) assays detecting the species-specific *tpi* gene, toxin A (*tcdA*) gene, toxin B (*tcdB*) gene, and the binary toxin (*cdtA/cdtB*) genes. Confirmed *C. difficile* isolates were tested against a panel of eight antimicrobials (vancomycin, metronidazole, rifampicin, ciprofloxacin, tetracycline, clindamycin, erythromycin, and ceftriaxone) using E-test strips.

**Results:**

*C. difficile* was detected in 57 (25%) of diarrheal patients over the age of two, 56 (98.2%) of whom received antimicrobials before the diarrheal episode. Amongst the 71 confirmed isolates, 69 (97.1%) harbored at least one toxin gene. More than half of the toxigenic isolates harbored a truncated *tcdA* gene. All isolates were sensitive to vancomycin, while three isolates (2.1%) were resistant to metronidazole (MIC >32 mg/L). High levels of resistance were observed to rifampicin (65/71, 91.5%), erythromycin (63/71, 88.7%), ciprofloxacin (59/71, 83.1%), clindamycin (57/71, 80.3%), and ceftriaxone (36/71, 50.7.8%). Among the resistant isolates, 61 (85.9%) were multidrug-resistant.

**Conclusion:**

Multidrug-resistant *C. difficile* strains were a significant cause of healthcare facility-onset *C. difficile* infections in patients with prior antimicrobial exposure in this Kenyan hospital.

## Introduction

*Clostridioides difficile* is an obligate anaerobic Gram-positive bacterium that colonizes 0 to 15% of the healthy human population (Furuya-Kanamori et al., 2015). However, the absence of a competitor gut microbiota as a result of antimicrobial therapy induces a shift from asymptomatic colonization to mild diarrhea that may progress to life-threatening diarrhea due to severe inflammation and perforation of the colon (Ferreyra et al., 2014; Pérez-Cobas et al., 2014). Substantial increases in the incidence and morbidity of healthcare facility-associated diarrhea attributed to *Clostridioides difficile* (previously named *Clostridium difficile*) (Lawson et al., 2016) during the past decade have resulted in recent attention being given to this pathogen.

In 2019, the United States Centers for Disease Control and Prevention (CDC) declared *C. difficile* as one of the five “urgent health threats” with the perception that it requires urgent and aggressive action because of the significant risks associated with antimicrobial overuse (CDC, 2019). Previous reviews reported a low and underestimated prevalence of *C. difficile* infection in low and middle-income countries. However, given that antimicrobial use is the primary driver of *C. difficile* infections, the frequency could be higher in countries where antimicrobial use is not regulated (Bebell and Muiru, 2014; Roldan et al., 2018). Testing for *C. difficile* infections in developing countries is not routinely carried out owing to a lack of resources for diagnostic testing and the culture facilities for obligate anaerobes. Therefore, diarrhea is treated symptomatically, leading to misdiagnosis, mistreatment, and a possible underestimation of the contribution of *C. difficile* to diarrhea.

*C. difficile* infection (CDI) is primarily attributed to the production of toxins A and B and occasionally binary toxins. While the majority of symptomatic disease, severe cases, and nosocomial outbreaks have been associated with strains that produce both toxin A and B (A^+^B^+^), new variants continue to emerge. Toxin A^−^B^+^ variants, such as those belonging to ribotype (RT) 017, harbor a truncation in the 3′-region of the toxin A gene (*tcdA*) that results in a TcdA negative phenotype. PCR primers such as those developed by Lemee et al. (2004) have been designed to amplify the partially deleted *tcdA* fragment allowing for correct characterization of the most common A^−^B^+^ variant strains (Lemee et al., 2004).

Prior antimicrobial exposure not only triggers the expansion of *C. difficile* populations in the gastrointestinal tract but also facilitates toxin production by the organism, cumulatively contributing to an approximately 60% increased risk of healthcare facility-onset CDI (HO-CDI) (Slimings and Riley, 2014). The fluoroquinolones, clindamycin, and cephalosporin antibiotics are known to trigger toxin-mediated CDI more than other classes of antimicrobials (Gerding, 2004; Slimings and Riley, 2014; Peng et al., 2017). Over the years, new variants of *C. difficile* resistant to these antimicrobials and with reduced susceptibility to first-line antimicrobials (metronidazole and vancomycin) against CDI have emerged (Spigaglia, 2016). High levels of resistance to clindamycin, ciprofloxacin, moxifloxacin, erythromycin, and imipenem especially among ribotypes 027 and 078 have largely been documented in Northern America, Europe, and Asia (Tenover et al., 2012; Keessen et al., 2013; Wieczorkiewicz et al., 2016).

In Kenya, recent studies have reported a CDI prevalence of 93.3% and 37.6% in young adults with diarrhea and children under the age of five, respectively, with the majority of the strains producing both toxins A and B (Oyaro et al., 2018; Plants-Paris et al., 2019). The findings were significantly higher than the 0% prevalence previously reported by Mwachari and colleagues two decades ago (Mwachari et al., 1998). This increased prevalence could be due to the differing diagnostic assays used and possibly, epidemiological changes following significant increases in CDI and emerging variants of *C. difficile.* Therefore, this study was conducted to determine the prevalence of CDI in hospitalized patients at Kenyatta National Hospital and to evaluate the antibiotic resistance and toxin profiles of the isolated *C. difficile* strains.

## Materials and Methods

### Study Setting and Patient Recruitment

A cross-sectional study was conducted among patients admitted at a large referral hospital in Nairobi Kenya, the Kenyatta National Hospital. The study participants included patients of all ages who developed diarrhea ≥3 days following hospitalization. From 2016 to 2018, a total of 333 patients were consecutively recruited in all in-patient clinical departments. To recruit patients, two approaches were employed. In the first approach, patients meeting the eligibility criteria (diarrhea onset >72 h after admission and ≥3 loose stools in 24 h preceding assessment) were identified from the patient’s files in the wards and were invited to participate and provide consent/assent. The second approach identified watery stool samples received in the laboratory and traced them back to patients in the wards to seek consent/assent if they met the inclusion criteria. For each patient that consented, data on the age, sex, date of admission, reason for admission, history of previous admission, admission ward, duration of diarrhea, antimicrobial exposure during the hospitalization period, and pre-existing comorbidities was collected. A unique identifying number was assigned to each study subject and used on the questionnaire and the stool collection container.

### Case Definition

*C. difficile* infection (CDI) was defined as the presence of unexplained clinical diarrheal symptoms (≥3 loose stools in 24 h) plus a positive nucleic acid amplification test (NAAT) for the *C. difficile tpi* gene, toxin A and/or toxin B, or the binary toxins. A case was considered a healthcare facility-onset *C. difficile* infection (HO- CDI) if the CDI symptoms occurred >3 days after admission.

### Culture and Isolation of *C. difficile*

The stool samples were alcohol-shocked in an equal volume of absolute ethanol. The suspension was then vortexed and allowed to stand at room temperature for 1 h. Following centrifugation of the sample at 4,000 rpm for 1 min, 50 µl of the sample was directly streaked onto cycloserine-cefoxitin egg yolk agar (CCEY) (LabM, United Kingdom) to obtain distinct colonies. The culture plates were then incubated anaerobically at 37°C for 48 h using anaerobic jars and anaerobic gas generating sachets (Oxoid, United Kingdom). *C. difficile* were presumptively identified based on the presence of gray, opaque, elongating, colonies with fimbriated edges and a typical phenolic odor and the appearance of Gram-positive rods on microscopy. Pure isolates were stored at −80°C in 1 ml of brain‐heart infusion (BHI) supplemented with 10% glycerol. *Clostridium difficile* DSMZ 27147 was used as a positive control strain (https://www.dsmz.de/catalogues/details/culture/DSM-27147.html).

### Confirmation of the Isolates

DNA extraction was performed on all presumptive *C. difficile* isolates using the Zymo Research Quick-DNA Fungal/Bacterial Miniprep Kit (Zymo Research, Irvine, CA, USA) with a final elution volume of 50 µl. We confirmed the identity of the presumptive isolates by testing for the *C. difficile*-specific triosephosphate isomerase (*tpi*) housekeeping gene in an in-house qPCR assay using the *tpi*-specific primer set designed by (Lemee et al., 2004). The qPCR amplification and analysis were performed with a Magnetic Induction Cycler with the micPCRv2.4.0 software (Bio Molecular Systems, Sydney, NSW, Australia). A 20 µl reaction was prepared by mixing 10 µl of 2X Luna Universal qPCR SYBR Green master mix (New England BioLabs, UK) with 7 µl of nuclease-free water, 0.5 µl of both forward and reverse primers (10 µM stock solutions), and 2 µl of template DNA. The temperature cycling parameters were as follows: one cycle of 95°C for 3min, followed by 40 cycles of 95°C for 30s, 60°C for 30s, and 72°C for 30s. A dissociation curve was generated post-amplification with a ramp from 72°C to 95°C to confirm the amplification specificity.

### Real-Time PCR Assay for Detection of Toxins *tcdA, tcdB*, and Binary Toxin (*cdtA, ctdB)*

Singleplex SYBR Green-based qPCR assays were carried out on all the *tpi* positive isolates to investigate the presence of the *C. difficile* toxin A (*tcdA*), toxin B (*tcdB*) genes, as well as the accessory toxins A (*cdtA*) and toxin B (*cdtB*). For each reaction, a final volume of 20 µl reaction mix was prepared to contain 10 µl of 2X Luna Universal qPCR master mix, 7 µl of nuclease-free water, 0.5 µl of both forward and reverse primers (10 µM stock solutions), and 2 µl of extracted DNA template. The temperature cycling parameters and melt curve analysis were identical to those used to screen for the *tpi* gene.

As noted previously (Lemee et al., 2004), the *tcdA* primers were designed to flank the smallest of the three deletions in the 3’ region of the gene generating a shorter fragment (110 bp) for the most common deleted form of the gene. The qPCR assay was able to distinguish strains with a truncated *tcdA* gene from those with a full-length gene by the associated melting profiles. Samples with a truncated *tcdA* gene had a significantly lower melting temperature than those with the full-length *tcdA*. A toxigenic strain of *C. difficile* was defined as an isolate harboring any one or a combination of the genes: *tcdA*, *tcdB*, and *cdtA/B*. *C. difficile* DSMZ-27147, which carries all these genes, was used as an internal control to validate the PCR results.

### Antimicrobial Susceptibility Testing

Pure colonies of *C. difficile* were diluted to a 0.5 McFarland standard and swabbed on Brucella Base agar supplemented with 5% sheep blood, vitamin K1 (0.5 mg/ml), and hemin (5 mg/ml) (Becton & Dickinson, USA). E-test strips (BioMérieux, France) for metronidazole, vancomycin, ciprofloxacin, erythromycin, clindamycin, tetracycline, rifampicin, and ceftriaxone were then placed on the culture plates. Following a 48 h incubation in anaerobic conditions, minimum inhibitory concentrations (MICs) for each of the antimicrobials tested were determined and interpreted as per the Clinical and Laboratory Standards Institute (CLSI, M100, 2020) and the European Committee on Antimicrobial Susceptibility Testing (EUCAST, version 10) guidelines. The MIC breakpoints were categorized into susceptible (S), intermediate (I), and resistant (R). The MIC breakpoints for all the antimicrobials tested except rifampicin were determined according to CLSI 2020. The EUCAST defined epidemiological cut-off value (ECOFF) of 0.004 mg/L was used to interpret the susceptibility of rifampicin. Both CLSI and EUCAST standards do not specify the breakpoints for ciprofloxacin so the CLSI breakpoints of moxifloxacin were used as a proxy. Multidrug-resistance (MDR) was defined as non‐susceptibility to at least one agent in three or more antimicrobial categories. *C. difficile* DSM 27147 (R20291) with published MICs (vancomycin [0.2–0.93 mg/L], metronidazole [0.21–0.9 mg/L], clindamycin [18 mg/L], erythromycin [≥256 mg/L], ciprofloxacin [≥32 mg/L], tetracycline [0.22 mg/L]) served as an internal control (Stabler et al., 2009; Mathur et al., 2013; Kelly et al., 2016).

### Statistical Analysis

The data were analyzed using SPSS version 21 and Microsoft Excel. Both continuous and categorical variables were summarized as frequencies and percentages of the study population. Two-sided Fisher’s exact tests were used to compare groups of continuous variables. Antibiograms were tabulated as proportions of isolates that were susceptible, intermediate, or resistant.

### Ethical Approvals

Ethical approval was sought from the Kenyatta National Hospital-University of Nairobi Ethics and Research Committee (P8/01/2014). Per ethical standards, the study objectives were explained to the participants in either English or Swahili and those willing to participate signed the informed consent/assent forms.

## Results

### Demographics and Admission Information

A total of 333 patients with diarrhea during the study period consented to participate. The baseline data for patients with and without CDI are summarized in **Table 1**. All the patients provided stool samples for microbiological analysis. There were more females, 170 (51.1%) than males, 163 (48.9%), with the majority of patients falling within the 26–45 years age range (108, 32.4%). Additionally, a relatively large number of patients (101, 30.3%) were below 2 years old at the time of sampling, and these were excluded from the subsequent prevalence and antimicrobial prescription analyses described below due to high *C. difficile* asymptomatic colonization. More than a third of the patients (137, 41.1%) had hospital stays of more than 4 weeks, and 48 (14.4%) of the patients reported a history of prior admission within the previous 3 months. Overall, 168 (50.5%) of the patients had infectious diseases with antimicrobial use before the onset of diarrhea, noted in 297 (89.2%) patients. More than half of the patients, 230 (69.1%), had comorbidities, including HIV, tuberculosis, anemia, diabetes, and hypertension. Of the 232 patients >2 years, 57 had CDI, giving an overall CDI prevalence of 24.6%. After stratifying based on patient age, the highest prevalence (31.6%) was observed for patients who were ≥60 years old, followed by those in the 3–15-year-old range (30.2%). The lowest prevalence was observed in patients between the ages of 16 and 25 years.

**Table 1:** Demographic and admission information of the study patients (n = 333).

	*C. difficile* positive patients (%) (n = 71)	*C. difficile* negative patients (%) (n = 262)	Total
Age group in years (mean = 24 years)*			
≤2	14 (13.9)	87 (86.1)	101 (30.3)
3–15	13 (30.2)	30 (69.8)	43 (12.9)
16–25	5 (16.1)	26 (83.9)	31 (9.3)
26–45	25 (23.1)	83 (76.9)	108 (32.4)
45–59	8 (26.7)	22 (73.3)	30 (9.0)
≥60	6 (31.6)	13 (68.4)	19 (5.7)
**Gender**			
Female	33 (46.5)	137 (52.3)	170 (51.1)
Male	38 (23.3)	125 (76.7)	163 (48.9)
**Admission ward**			
Internal medicine	27 (38.0)	91 (34.7)	118 (35.4)
Pediatric	26 (36.7)	90 (34.4)	116 (34.8)
Surgical	14 (19.7)	45 (17.2)	59 (17.7)
Other	4 (5.6)	36 (13.7)	40 (12.0)
**Duration of diarrhea**			
<1 week	55 (77.5)	228 (87.0)	283 (85.0)
1–3 weeks	14 (19.7)	29 (11.1)	43 (12.9)
>3 weeks	2 (2.8)	5 (1.9)	7 (2.1)
**Antimicrobial use**			
Yes	70 (98.6)	227 (86.6)	297 (89.2)
No	1 (1.4)	35 (13.4)	36 (10.8)
**Previous admission in the last 3 months**			
Yes	20 (28.2)	28 (10.7)	48 (14.4)
No	51 (71.8)	234 (89.3)	285 (85.6)
**Duration of hospitalization**			
≤1 week	8 (12.7)	55 (87.3)	63 (18.9)
2 weeks	15 (18.1)	68 (81.9)	83 (24.9)
3 weeks	13 (26.0)	37 (74.0)	50 (15.01)
≥4 weeks	35 (25.5)	102 (74.5)	137 (41.1)
**Reason for hospitalization**			
Infectious diseases	44 (62.0)	124 (47.3)	168 (50.5)
Non-Infectious diseases	27 (38.0)	138 (52.7)	165 (49.5)
**Comorbidity**			
Yes	63 (88.7)	167 (63.7)	230 (69.1)
No	8 (11.3)	95 (36.3)	103 (30.9)

*Percentages in patient age categories are calculated as a proportion of patients in each category.

### Isolation and Toxin Profile of *C. difficile*

**Figure 1:**
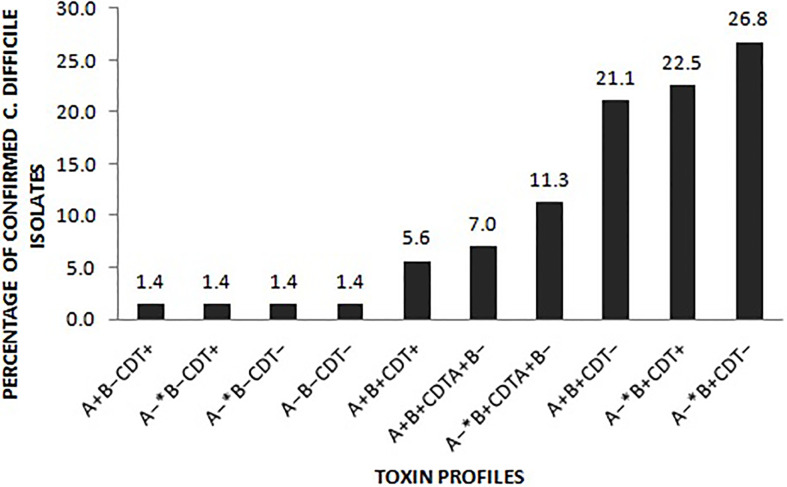
Distribution of the toxin types of the 71 C*. difficile* isolates. A^−^ *: isolates in which the *tcdA* gene had a 110 bp deletion compared to the wildtype *tcdA* gene. CDT^+^: isolates harboured both *cdtA* and *cdtB* gene of the binary toxin. CDTA^+^B^−^: isolates only harboured the A component of the binary toxin. CDT^–^ isolates lacked either of the binary toxin genes.

**Figure 2:**
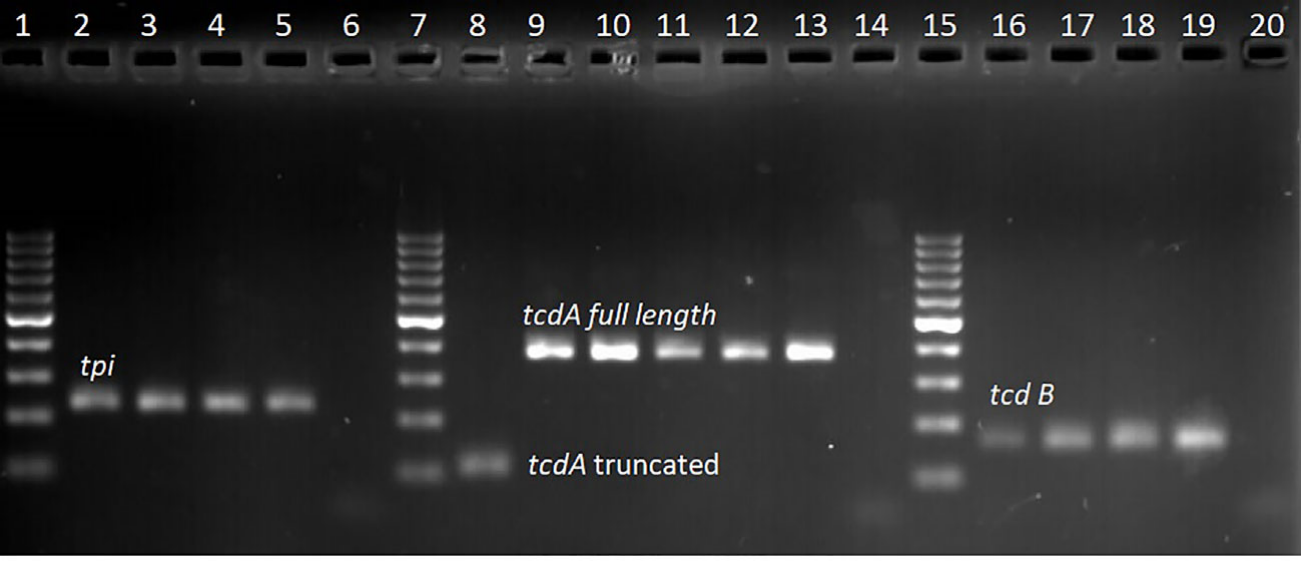
Gel electrophoresis of qPCR amplicon products; lanes 1, 7, and 15 represent 100 bp molecular weight marker, lanes 2–4 *tpi* positive (230 bp) clinical isolates, lane 5 *tpi* positive control (*C. difficile* DSM 27147), lane 6 no template control. Lane 8 clinical isolate with truncated *tcdA* (110 bp) amplicon, lane 9–12 clinical isolates with full length *tcdA* gene (369 bp), lane 13 positive control (*C. difficile* DSM 27147), and lane 14 no template control. Lane 16–18 clinical samples with *tcdB* gene (160 bp), lane 19 positive control (*C. difficile* DSM 27147), and lane 20 no template control.

Of the 333 stool samples cultured on CCEY, *C. difficile* was recovered from 71 (21%) samples, all of which tested positive for the *tpi* gene. Of the 71 confirmed isolates, 69 (97.1%) and 2 (2.8%) were toxigenic and non-toxigenic *C. difficile* isolates, respectively. The toxigenic isolates comprised of 4 (5.6%) with toxin A, toxin B, and binary toxin (A^+^B^+^ CDT^+^), 15 (21.1%) isolates with both toxin A and toxin B but devoid of binary toxin (A^+^B^+^ CDT^−^), 16 (22.5%) positive for toxin B and binary toxin with a 110 bp deletion in the *tcdA* gene (A^−*^B^+^ CDT^+^) while 19 (26.8%) isolates were positive for truncated toxin A, toxin B but lacked the binary toxin (A^−*^B^+^ CDT^−^). Two isolates did not express *tcdB* gene one of which was positive for the full-length *tcdA* gene and *cdtAB* genes (A^+^B^−^CDT^+^) while the other had a 110 bp deletion in the *tcdA* gene but harboured the *cdtAB* genes (A^−*^B^−^CDT^+^). We also observed isolates that only harboured the A component of the binary toxin; 5 (7.0%) of A^+^B^+^ CDTA^+^/CDTB^−^, and 8 (11.3%) of A^−*^B^+^ CDTA^+^/CDTB^−^. The two non-toxigenic isolates comprised 1 (1.4%) isolate which appeared to harbor a truncated *tcdA* gene but lacked the *tcdB* genes and the binary toxin (A^−*^B^−^CDT^−^) and the other that completely lacked the PaLoc as well as the *cdt* genes despite multiple attempts to amplify them. The distribution of toxigenic and non-toxigenic types of *C. difficile* strains is summarized in **Figure 1** with a representative gel electrophoresis image in **Figure 2**.

### Comparison of Antimicrobial Use Between Patients With and Without *C. difficile*

Antimicrobial use before the onset of diarrhea was noted in 197 (84.9) of the patients who were 2 years of age or older. Ceftriaxone 121 (52.2), amoxicillin/clavulanic acid (69, 29.7%), and metronidazole (73, 31.5%) were the most commonly prescribed antimicrobials. Antimicrobial use differed significantly in patients who tested positive for *C. difficile* from those who tested negative (**Table S1** in supplementary material). Patients with CDI were more likely to have received any antimicrobials (p = <0.001), and significant associations were seen for amoxicillin/clavulanic acid, penicillins, ceftriaxone, meropenem, amikacin, clarithromycin, ciprofloxacin, cotrimoxazole, clindamycin, and anti-TB drug use.

### Antimicrobial Susceptibility of the *C. difficile* Isolates

All 71 isolates (100%) were sensitive to vancomycin (MIC ≤2 µg/ml). Varying proportions of resistance were noted for all the other antimicrobials. High frequencies of resistance to rifampicin (65, 91.5%), erythromycin (63, 88.7%), ciprofloxacin (59, 83.1%), clindamycin (57, 80.3%), and ceftriaxone (36, 50.7%) were observed. In contrast, resistance to metronidazole and tetracycline was observed for only 3 (4.2%) and 7 (9.9%) of the isolates, respectively. The antibiogram profile for each of the antimicrobials tested is presented in **Table 2**.

**Table 2:** Antimicrobial susceptibility pattern of *C. difficile* isolates (n = 71).

Antimicrobial agent	MIC range (mg/L)	MIC breakpoint criteria (mg/L)				Frequency (%)		
		S	I	R	ECOFF	S	I	R
Vancomycin^a^	0.125–0.5	≤2		≥4		71 (100)	–	0 (0)
Metronidazole^a^	0.016–256	≤8	16	≥32		68 (95.8)	0 (0)	3 (4.2)
Clindamycin^a^	0.023–256	≤2	4	≥8		13 (18.3)	1 (1.4)	57 (80.3)
Cefriaxone^a^	0.064–256	≤16	32	≥64		22 (31.0)	13 (18.3)	36 (50.7)
Erythromycin^a^	0.094–256	≤2	4	≥8		8 (11.3)	–	63 (88.7)
Rifampicin^b^	0.002–32				0.004	6 (85.0)	–	65 (91.5)
Ciprofloxacin^a*^	0.064–32	≤2	4	≥8		10 (14.1)	2 (2.8)	59 (83.1)
Tetracycline^a^	0.016–32	≤4	8	≥16		35 (49.3)	29 (40.8)	7 (9.9)

^a^Breakpoints per CLSI MIC values for anaerobes. ^b^Breakpoints per EUCAST MIC guidelines for C. difficile. *The Moxifloxacin breakpoint is used as a proxy. MIC, minimum inhibitory concentration; S, susceptible; I, intermediate; R, resistant; ECOFF, epidemiological cut-off value.

### Multiple Antimicrobial Resistance

Among the 71 C*. difficile* isolates, 70 (98.6%) isolates were resistant to at least one antimicrobial, while 68 isolates (95.7%) displayed resistance to more than two classes of antimicrobials. Based on the multiple antimicrobial resistance analysis, 61 isolates (85.9%) were multidrug-resistant, showing resistance to more than three classes of antimicrobials. Twenty-three isolates (37.7%) showed resistance to more than six antimicrobials, while the majority, 26 (42.6%), were resistant to five antimicrobials. There was a tendency for slightly higher proportions of A^+^B^+^ CDT^−^ isolates to show resistance to the majority of the tested antimicrobials, although the difference was not statistically significant (**Table 3**).

**Table 3:** Comparison of antimicrobial resistance by toxin variant types of *C. difficile* strains.

Resistance phenotype	Toxigenic strains								Nontoxigenic strains		*p-*value*^#^*
	*A^+^B^+^ CDT^+^*(n = 4)	*A^+^B^+^ CDT^−^*(n = 15)	*A^+^B^+^ CDTA^+^B^−^*(n = 5)	*A^+^B^−^ CDT^+^*(n = 1)	A^−*^B^+^CDT^+^(n = 16)	A^−*^B^+^ CDT^−^(n = 19)	A^−*^B^+^ CDTA^+^B^−^(n = 8)	A^−*^B^−^CDT^+^(n = 1)	A^−*^B^−^CDT^−^(n = 1)	*A^−^B^−^CDT^−^*(n = 1)	
Metronidazole (n = 3)	–	1 (6.7)	–	–	2 (12.5)	–	–	–	–	–	1.000
Clindamycin (n = 58)	4 (100)	12 (80.0)	3 (60.0)	1 (100)	16 (100)	14 (73.7)	7 (87.5)	–	–	1 (100)	1.000
Ceftriaxone (n = 49)	2 (50.0)	9 (60.0)	2 (40.0)	1 (100)	13 (81.3)	13 (68.4)	7 (87.5)	1 (100)	–	1 (100)	0.724
Erythromycin (n = 63)	3 (75.0)	14 (93.3)	4 (80.0)	1 (100)	16 (100)	16 (84.2)	7 (87.5)	–	1 (100)	1 (100)	0.613
Rifampicin (n = 65)	3 (75.0)	15 (100)	3 (60.0)	1 (100)	16 (100)	17 (89.5)	8 (100)	–	1 (100)	1 (100)	0.492
Ciprofloxacin (n = 61)	4 (100)	13 (86.7)	4 (80.0)	1 (100)	15 (93.8)	13 (68.4)	8 (100)	1 (100)	1 (100)	1 (100)	0.257
Tetracycline (n = 36)	2 (50.0)	9 (60.0)	3 (60.0)	1 (100)	11 (73.3)	5 (26.3)	4 (50.0)	–	–	1 (100)	0.080
MDR (n = 61)	3 (75.0)	14 (93.3)	3 (60.0)	1 (100)	16 (100)	14 (73.7)	8 (100)	–	1 (100)	1 (100)	0.196
**MDR patterns**											
Three classes (n = 6)	–	4 (26.7)	–	–	–	–	1 (12.5)	–	1 (100)	–	0.030
Four classes (n = 6)	–	2 (13.3)	1 (20.0)	–	1 (6.3)	2 (10.5)	–	–	–	–	1.000
Five classes (n = 26)	1 (25.0)	5 (33.3)	2 (40.0)	–	6 (37.5)	8 (42.1)	4 (50.0)	–	–	–	0.728
≥Six classes (n = 23)	1 (25.0)	4 (26.7)	–	1 (100)	9 (56.3)	4 (21.1)	3 (37.5)	–	–	1 (100)	0.709

A**^−^** ^*^: isolates in which the tcdA gene had a 110 bp deletion compared to the wildtype tcdA gene. CDT^+^: isolates harboured both cdtA and cdtB genes of the binary toxin. CDTA^+^B^−^: isolates only harboured the A component of the binary toxin. **^#^**P-value the result of a two-tailed Fishers exact test comparing A^+^B^+^CDT^−^ isolates to A^−^*B^+^CDT^−^ isolates. MDR, Multidrug-resistant.

## Discussion

Data on the prevalence of CDI in sub-Saharan Africa are currently lacking. In this study, we determined a CDI prevalence of 24.6% in hospitalized patients with healthcare facility-onset diarrhea in a large hospital in Kenya. The majority of these patients were previously exposed to antimicrobials such as ceftriaxone, amoxicillin/clavulanic acid, and metronidazole. Many of the *C. difficile* strains isolated from this patient population were highly resistant to rifampicin, clindamycin, erythromycin, ciprofloxacin, and ceftriaxone, and a large proportion were multidrug-resistant. Similar findings where more than half of the *C. difficile* isolates recovered were MDR have been associated with outbreaks (Obuch-Woszczatyński et al., 2014; Krutova et al., 2015; Spigaglia, 2016; Ramírez-Vargas et al., 2017; Carman et al., 2018; Zhou et al., 2019). These findings highlight the significance of this pathogen in an African population previously considered to be at low CDI risk (Roldan et al., 2018). Published data from studies conducted in Africa show that *C. difficile* prevalence varies from as low as 0% to as high as 93% depending on the population sampled and the testing method used (Mwachari et al., 1998; Zulu et al., 2000; Onwueme et al., 2011; Beadsworth et al., 2014; Simango and Uladi, 2014; Kullin et al., 2015; Seugendo et al., 2015; Janssen et al., 2016, Oyaro et al., 2018). These variations make it difficult to directly compare studies. Nevertheless, the prevalence rate for *C. difficile* in diarrheal patients in our setting is within the range generally reported for studies internationally, where *C. difficile* is thought to be responsible for 15–25% of antibiotic-associated diarrheas (Bartlett and Gerding, 2008).

Infants under the age of 2 years old are typically excluded from *C. difficile* surveillance studies due to the high prevalence of asymptomatic colonization in this group (McDonald et al., 2018). For this reason, in our study, we excluded these patients from our epidemiological analyses. However, we chose to include isolates from infants in our toxin profiling and antimicrobial susceptibility testing analyses due to the potential for infants to serve as reservoirs of clinically relevant strains of *C. difficile* (Rousseau et al., 2012; McLure et al., 2019). Interestingly, while the prevalence of CDI in older patients (≥60 years) was slightly higher than in the other age groups in our setting, there did not appear to be a strong association of CDI with age that is typically observed in European and US studies. This disparity has been noted in African studies of CDI and may reflect the generally younger African population as well as differences in underlying risk factors (Rajabally et al., 2013).

While almost all isolated strains were PCR-positive for at least one toxin, one isolate did not express either of the toxin genes Notably, the majority of our isolates were A^−*^B^+^variants harboring a previously described truncated version of the *tcdA* gene (Lemee et al., 2004) different from the association with A^+^B^+^ variants previously described (Oyaro et al., 2018). A^−*^B^+^ variants are particularly common in Asia but have been identified in many countries across the world. It is thought that the early reliance on diagnostic tests that only targeted toxin A allowed A^−*^B^+^ strains to circulate undetected for an extended period, facilitating their spread (Imwattana et al., 2019). Importantly, A^−*^B^+^ strains are capable of causing severe and recurrent disease and have been associated with several major nosocomial outbreaks in Dublin (Drudy et al., 2007), Canada (Al-Barrak et al., 1999), Australia (Elliott et al., 2011), Japan (Sato et al., 2004), Israel (Samra et al., 2002), and Netherlands (Kuijper et al., 2001) predominantly belonging to ribotype (RT) 017. The appearance of strains expressing the binary toxin gene in Kenyan hospitalized patients is noteworthy considering that they have previously been linked to increased disease severity (Gerding et al., 2014). Therefore, further studies are needed to determine whether the high prevalence of A^−*^B^+^ strains is due to a localized outbreak or simply reflects the pattern of strains more generally found in the region.

Interestingly, three of the strains isolated in the current study exhibited the rare A^+^B^−^ variation. All three of these were PCR positive for the *tcdA* gene (full length and truncated) but failed to yield a product for the *tcdB* gene, despite several attempts. An important limitation of the *tcdA* primer set employed in this study is that it does not allow the identification of additional deletions within the 5’- region of the gene, such as those present in toxinotype XI strains (Geric Stare and Rupnik, 2010). While true A^+^B^−^ variants are rare, they have been reported previously (Monot et al., 2015), and it would be interesting to examine the pathogenicity locus further in these isolates. Similarly some of the isolated strains also harbored *cdtA* and not *cdtB*. These strains are unusual however it is not the first time that they have been reported (Azimirad et al., 2018).

The burden of HIV/AIDS and TB, together with infections by multidrug-resistant organisms (MDRO) in Africa, has increased the number of individuals requiring prolonged hospitalization (Serra-Burriel et al., 2020; Singh et al., 2020). Long stays in hospitals can lead to inappropriate and prolonged use of antimicrobials, gradually increasing the risk of acquiring *C. difficile* infections (Onwueme et al., 2011; Seugendo et al., 2015; Kullin et al., 2018). In the current study, most of the patients were admitted for infectious diseases accounting for the high levels of antimicrobial use among the study participants. In particular, we observed the predominant use of ceftriaxone, amoxicillin/clavulanic acid and metronidazole similar to what has been documented in Kenya, Tanzania, Uganda, and other countries that participated in the Global Point Prevalence Survey (Global-PPS) (Kiguba et al., 2016; Versporten et al., 2018; Momanyi et al., 2019; Sonda et al., 2019). Antimicrobial prescription decisions in many facilities in resource-limited settings are primarily empirical due to the relatively high costs of laboratory investigations and a lack of facilities to perform anaerobic culture precluding specific diagnostic and antimicrobial susceptibility testing (Chem et al., 2018). We also noted that the majority of the patients were taking more than one antimicrobial agent drug. Simultaneous treatment with multiple antimicrobials has a more profound effect on the indigenous gut microbiota and promotes the spread of *C. difficile* more than monotherapy.

In a recent review by (Spigaglia, 2016), resistance to clindamycin, fluoroquinolones, cephalosporins, and erythromycin was common among clinical isolates of *C. difficile*. This resistance profile was mirrored in the isolates in this study, which had relatively high frequencies of resistance to the macrolide-lincosamide-streptogramin B (MLS_B_) family of antimicrobials (clindamycin, erythromycin), fluoroquinolone (ciprofloxacin), cephalosporins (ceftriaxone), and rifamycins (rifampicin). The clindamycin, fluoroquinolone, and cephalosporin groups of antimicrobials are known to be associated with an increased risk of developing CDI. For example, the acquisition of high-level resistance to fluoroquinolones is thought to be associated with major outbreaks of the “hypervirulent” *C. difficile* 027/BI/NAP1 strains (He et al., 2013; Spigaglia, 2016). Given the high rates of antimicrobial consumption in our setting, it is not surprising that many of the isolates showed increased levels of resistance. Notably, for antimicrobials such as rifamycins and fluoroquinolones, the resistance mutations often come without a fitness cost to the organism and are, therefore, stably maintained in the population (Wasels et al., 2015). Additionally, the resistance of *C. difficile* to MLS_B_ class antimicrobials and tetracyclines is mainly mediated by *ermB* and *tet* genes, respectively, both of which are usually present in mobile genetic elements that promote the horizontal transfer of resistance between strains (Mullany et al., 2015).

Previously, prolonged rifampicin use, especially in the treatment of TB, was implicated in the emergence of rifampicin resistance in *C. difficile* (Choi et al., 2011). Kenya is listed by the World Health Organization (WHO) as one of the 30 high burden TB countries (Enos et al., 2018). Therefore, the high resistance (91.5%) of *C. difficile* isolates to rifampicin could be as a result of selective pressure following extensive use of rifampicin in first-line treatment regimens for TB. The results from this study, therefore, build on findings from a recent study in Cape Town, South Africa that found a very high level of rifampicin resistance (~95% of strains resistant) in *C. difficile* from patients undergoing TB treatment (Kullin et al., 2018).

Metronidazole and vancomycin remain the first choice antimicrobials for treating *C. difficile* infections, even though reduced susceptibilities to these antimicrobials have been reported (Cohen et al., 2010). While almost all the isolates in the current study were susceptible to metronidazole, three strains had MICs of ≥32 mg/L. Recent reports of metronidazole treatment failures due to substantial prolonged exposure to the antimicrobial are on the rise (Spigaglia, 2016). Interestingly in this study, none of the three patients from whom these metronidazole resistant isolates were recovered had any prior metronidazole exposure. Metronidazole resistance mainly occurs as a result of alterations in metabolic pathways involved in DNA repair, iron metabolism, and the carriage of nitroreductases (Chong et al., 2014). Boekhoud et al. recently revealed that metronidazole resistance in *C. difficile* could be a result of a transmissible plasmid (Boekhoud et al., 2020). We however did not explore the mechanisms for antimicrobial resistance.

We noted that both the toxigenic and non-toxigenic variants in our setting tended to be resistant to the antimicrobials tested, although among the predominant variants (A^+^B^+^CDT^−^ and A^−^*B^+^CDT^−^) the differences in the resistance prevalences were not statistically significant. RT 017 strains are the most commonly described A^−^B^+^ isolates worldwide, and strains belonging to this ribotype have been reported to have a stronger association with MDR in several studies (Goorhuis et al., 2011; Spigaglia et al., 2011; Kim et al., 2016; Putsathit et al., 2017). Other important ribotypes associated with MDR include RT 027, RT 078, and RT 018 (Spigaglia, 2016). The reasons for these associations are not yet known. However, antimicrobial resistance has likely facilitated the spread of these isolates in the regions where they are found.

In conclusion, our data show that *C. difficile* is a clinically relevant pathogen in patients receiving in-patient services at Kenyatta National Hospital. Furthermore, antimicrobial resistance in *C. difficile* in our setting is potentially a serious problem that may result in untreatable infections or reliance on last-line expensive antimicrobials like vancomycin, therefore highlighting the need for enhanced antimicrobial stewardship programs. *C. difficile* should be considered in routine diagnosis of diarrhea cases to promote the development and implementation of strategies that regulate antimicrobials known to induce HA-CDI. As this is the first study to report the occurrence of MDR *C. difficile* strains in this region, future work should be undertaken to examine the diversity of strain types responsible for the disease (e.g., by ribotyping isolates) and correlate the disease with predisposing risk factors other than antimicrobial exposure.

## Data Availability Statement

The original contributions presented in the study are included in the article/**Supplementary Material**. Further inquiries can be directed to the corresponding author.

## Ethics Statement

This study involved human participants and was reviewed and approved by Kenyatta National Hospital-University of Nairobi Ethics and Research Committee. The patients/participants provided their written informed consent to participate in this study.

## Author Contributions

WM, MM, OA, and GR conceptualized and designed the study. WM and MB collected the samples and performed culture, identification, and antimicrobial susceptibility testing of the isolates. WM, LM, BK, CK, EO, and PO developed protocols for the molecular assays, performed, and interpreted the qPCR assay. LM, GR, and MM supervised laboratory experiments. WM analyzed the data and drafted the manuscript. BK, LM, and MM edited and revised the manuscript. All authors contributed to the article and approved the submitted version.

## Funding

WM was supported by the Consortium for Advanced Research Training in Africa (CARTA). CARTA is jointly led by the African Population and Health Research Center and the University of the Witwatersrand and funded by the Carnegie Corporation of New York (Grant No.: B 8606.R02), Sida (Grant No.: 54100029), the DELTAS Africa Initiative (Grant No: 107768/Z/15/Z). The DELTAS Africa Initiative is an independent funding scheme of the African Academy of Sciences (AAS) ‘s Alliance for Accelerating Excellence in Science in Africa (AESA) and supported by the New Partnership for Africa’s Development Planning and Coordinating Agency (NEPAD Agency) with funding from the Wellcome Trust (UK) (Grant No.: 107768/Z/15/Z) and the UK government. This work was also partially supported by funds from National Research Fund Kenya (NRF) (2017/2018), University of Nairobi Deans Research Grant (2015/2016), and AstraZeneca (2014/2015). The funder bodies were not involved in the study design, collection, analysis, interpretation of data, the writing of this article or the decision to submit it for publication.

## Conflict of Interest

The authors declare that the research was conducted in the absence of any commercial or financial relationships that could be construed as a potential conflict of interest.
