# Supplementary material for: High Prevalence of Multidrug-Resistant Clostridioides difficile Following Extensive Use of Antimicrobials in Hospitalized Patients in Kenya
Source: Front Cell Infect Microbiol. 2021 Feb 8;10:604986. doi: 10.3389/fcimb.2020.604986 (PMC7897694; doi:10.3389/fcimb.2020.604986)
Supplement: Supplementary file 1 [file Table_1.pdf]

**Table S1. Antimicrobial use in patients (>2 years old) with and without *C. difficile***

| Parameters | *C. difficile* positive (n=57) | *C. difficile* negative (n=175) | Total (n=232) | *P*-value |
|---|---|---|---|---|
| Antimicrobial exposure | 56 (98.2) | 141 (80.5) | 197 (84.9) | <0.001# |
| **Antimicrobial** | | | | |
| **Penicillins** | | | | |
| Amoxicillin/clavulanic acid | 24 (42.1) | 45 (25.7) | 69 (29.7) | 0.029# |
| Penicillins | 8 (14.0) | 7 (4.0) | 15 (6.6) | 0.013# |
| Flucloxacillin | 3 (5.3) | 6 (3.4) | 9 (3.9) | 0.693 |
| **Cephalosporins** | | | | |
| Ceftazidime | 4 (7.0) | 7 (4.0) | 11 (4.7) | 0.471 |
| Cefuroxime | 1 (1.8) | 5 (2.9) | 6 (2.6) | 1.000 |
| Ceftriaxone | 40 (70.2) | 81 (46.3) | 121 (52.2) | 0.002# |
| **Carbapenem** | | | | |
| Meropenem | 12 (21.1) | 15 (8.6) | 27 (11.6) | 0.016# |
| **Aminoglycosides** | | | | |
| Gentamicin | 8 (14.0) | 12 (6.9) | 20 (8.6) | 0.106 |
| Amikacin | 7 (12.3) | 5 (2.9) | 12 (5.2) | 0.011# |
| **Macrolides** | | | | |
| Azithromycin | 2 (3.5) | 1 (0.6) | 3 (1.3) | 0.150 |
| Clarithromycin | 7 (12.3) | 4 (2.3) | 11 (4.7) | 0.006# |
| Erythromycin | 1 (1.4) | 2 (1.1) | 3 (1.3) | 0.573 |
| **Fluoroquinolones** | | | | |
| Ciprofloxacin | 15 (26.3) | 17 (9.7) | 32 (13.8) | 0.003# |
| **Glycopeptides** | | | | |
| Vancomycin | 5 (8.8) | 7 (4.0) | 12 (5.2) | 0.174 |
| **Tetracycline** | | | | |
| Tetracycline | 1 (1.8) | 0 (0) | 1 (0.4) | 0.246 |
| Doxycycline | 0 (0) | 1 (0.6) | 1 (0.4) | 1.000 |
| **Sulfonamides** | | | | |
| Cotrimoxazole | 15 (26.3) | 22 (12.6) | 37 (16.0) | 0.021# |
| **Nitroimidazole** | | | | |

| | | | | |
|---|---|---|---|---|
| Metronidazole | 20 (35.1) | 53 (30.3) | 73 (31.5) | 0.514 |
| **Oxazolidinone** | | | | |
| Linezolid | 2 (3.5) | 1 (0.6) | 3 (1.3) | 0.150 |
| **Lincosamide** | | | | |
| Clindamycin | 9 (15.8) | 6 (3.4) | 15 (6.6) | 0.003# |
| **Anti-TB medication*** | 14 (24.6) | 18 (10.3) | 32 (13.8) | 0.013# |
| **No. of antimicrobials used** | | | | |
| One | 4 (7.0) | 40 (22.9) | 44 (19.0) | 0.006# |
| Two | 10 (17.5) | 55 (31.4) | 65 (28.0) | 0.043# |
| Three | 15 (26.3) | 21 (12.0) | 36 (15.5) | 0.019# |
| ≥ Four | 27 (47.4) | 23 (13.1) | 50 (21.6) | 0.000# |
| **Duration of antimicrobials** | | | | |
| ≤ 1 week | 7 (12.3) | 29 (16.6) | 36 (15.5) | 0.531 |
| 2 weeks | 11 (19.3) | 38 (21.7) | 49 (21.1) | 0.852 |
| 3 weeks | 10 (17.5) | 17 (9.7) | 27 (11.6) | 0.151 |
| ≥ 4 weeks | 28 (49.1) | 57 (32.6) | 85 (36.6) | 0.027# |

***** *Anti-TB medication included rifampicin, isoniazid, ethambutol, and pyrazinamide; # Significant P-values ≤0.05*
